# Scalp Acupuncture Protects Against Neuronal Ferroptosis by Activating The p62-Keap1-Nrf2 Pathway in Rat Models of Intracranial Haemorrhage

**DOI:** 10.1007/s12031-021-01890-y

**Published:** 2021-08-17

**Authors:** Ming-Yue Li, Xiao-Hong Dai, Xue-Ping Yu, Wei Zou, Wei Teng, Peng Liu, Xin-Yang Yu, Qi An, Xin Wen

**Affiliations:** 1 Department of Neurology, First Affiliated Hospital, Heilongjiang University of Chinese Medicine, Harbin, Heilongjiang Province China; 2 Heilongjiang University of Chinese Medicine, Harbin, Heilongjiang Province, China; 3 Clinical Key Laboratory of Integrated Traditional Chinese and Western Medicine, Heilongjiang University of Chinese Medicine, Harbin, Heilongjiang Province, China

**Keywords:** Intracerebral haemorrhage, Scalp acupuncture, Ferroptosis, p62, Nrf2, GPX4, FTH1

## Abstract

Intracerebral haemorrhage (ICH) can be a catastrophic event; even if the initial stages of the pathology were well-managed, a number of patients experience varied residual neurological deficits following the insult. Ferroptosis is a recently identified type of cell demise which is tightly linked to the neurological impairment associated with ICH. In the current work, the prophylactic impact of scalp acupuncture (SA) therapy on autologous blood injection murine models of ICH was investigated in order to establish whether SA could mitigate the secondary damage arising following ICH by moderating ferroptosis. The pathophysiological mechanisms associated with this process were also explored. Ludmila Belayev tests were utilised for the characterisation of neurological damage. Haematoxylin–eosin staining was employed in order to determine the cerebral impact of the induced ICH. Malondialdehyde (MDA) and iron titres in peri-haemorrhagic cerebral tissues were appraised using purchased assay kits. Transmission electron microscopy delineated mitochondrial appearances within nerve cell bodies from the area of haemorrhage. Western blotting techniques were utilised to assay the degree of protein expression of NeuN, sequestosome 1 (p62), nuclear factor erythroid 2-related factor 2 (Nrf2), Kelch-like ECH-associated protein 1 (Keap1), glutathione peroxidase 4 (GPX4) and ferritin heavy chain 1 (FTH1). The frequencies of Nrf2, GPX4 and FTH1 positive cells, respectively, were documented with immunohistochemical staining. The results demonstrated that therapy with SA after ICH mitigated MDA and iron sequestration, diminished the appearance of contracted mitochondria with increased outer mitochondrial membrane diameter within the nerve cell bodies, and suppressed neuronal ferroptosis. The pathways responsible for these effects may encompass amplified p62, Nrf2, GPX4 and FTH1 expression, together with decreased Keap1 expression. Application of SA reduced identified neurobehavioural abnormalities after ICH; no disparities were observed between the consequences of SA therapy and deferoxamine delivery. It can be surmised that intervention with SA enhanced recovery after ICH by triggering the antioxidant pathway, p62/Keap1/Nrf2, and causing FTH1 and GPX4 upregulation, factors that participate in diminishing excess iron and thus in mitigating lipid peroxidation insults arising from ferroptosis following ICH.

**Supplementary Information:**

The online version contains supplementary material available at 10.1007/s12031-021-01890-y.

## Introduction

As one of the major life-threatening human pathologies, intracerebral haemorrhage (ICH) is the aetiology underpinning approximately 8–15% of cerebrovascular accidents in the United States each year (Unnithan and Mehta 2021). Worldwide epidemiological statistics have indicated that 24.6 individuals per 100,000 experience ICH; these figures are even greater in Asian communities (Weimar and Kleine-Borgman 2017; Haller et al. 2019). The rising incidence of ICH is thus an area of global concern (Sedova et al. 2021).

Death rates from ICH range from 25–30% in developed nations but are 5–18% greater in less affluent states. It is possible to decompress the brain and to evacuate blood clots through operative interventions; haemostatic pharmaceutical agents, rigorous blood control and additional management strategies can also be utilised to attain impressive clinical outcomes following ICH. Nevertheless, there are risks to these approaches. The long-term advantages of tranexamic acid for the control of bleeding are not fully elucidated, and chronic utilisation of VII chain A (RF chain A) may increase the likelihood of thrombotic events (Mayer et al. 2008). Additionally, 80% of individuals suffering an ICH experience residual altered levels of consciousness, hemiplegia or other neurological impairments (Rocha et al. 2020). The attention of research studies has therefore been drawn towards developing methods to circumvent the demise of multiple cerebral cells and to diminish the consequent morbidity impact of ICH. In particular, contemporary studies have focused on the detailed analysis of iron metabolism alterations and antioxidant influences following ICH, together with ways in which to defer neuronal death by moderating intracellular metabolism and stimulating antioxidant factors. These approaches may offer potential aids to rehabilitation following this haemorrhagic cerebral insult.

Lately, several research groups have demonstrated that Fe^2+^-dependent lipid peroxidation distinguishes ferroptosis from other methods of governed cell death (Tang et al. 2021; Buccarelli et al. 2018; Müller et al. 2017). Evidence is amassing which connects the pathways underlying ferroptosis to neurological pathologies such as Alzheimer’s disease, Parkinson’s disease, Huntington’s disease and amyotrophic lateral sclerosis (Zille et al. 2019; Devos et al. 2019; Masaldan et al. 2019; De Gregorio-Rocasolano et al. 2019). Furthermore, data from experiments have implied that ferroptosis also arises in haemorrhagic cerebrovascular accidents (Bai et al. 2020; Li et al. 2017a, b, c; Zhang et al. 2018a, b; Guo et al. 2020). It was demonstrated that surplus quantities of iron were released following the degradation of haemoglobin after the development of an intracerebral haematoma. As a consequence, impaired mitochondria produced numerous reactive oxygen species (Lemasters 2017); the resultant interruption in glutamine metabolism gave rise to dysfunction within antioxidant pathways and hydroxyl radical synthesis which, in turn, precipitated nerve cell ferroptosis (Imai et al. 2017; De Gregorio-Rocasolano et al. 2019; Magtanong and Dixon 2018).

The p62/Keap1/Nrf2 pathway is a major actor in safeguarding cells from oxidative injury provoked by ferroptosis (Sun et al. 2016a, b, c; Liu et al. 2016). Research has demonstrated that this mechanism contributes to the governance of iron homeostasis and the reaction to antioxidants (O’Connell and Hayes 2015). Following contact with erastin, a compound which activates ferroptosis, the expression of p62 promotes both the nuclear accretion of nuclear factor erythroid 2-related factor 2 (Nrf2) and consequent ferritin heavy chain 1 (FTH1) and heme oxygenase-1 (HO1) transcription through the suppression of Kelch-like ECH-associated protein 1 (Keap1) stimulation (Cloer et al. 2018). FTH1 is able to stockpile surplus iron within cells and thus diminishes iron-related peroxidative injury; HO1 is an essential antioxidant enzyme (Imam et al. 2017; Sun et al. 2016a, b, c). Moreover, genes that encode for proteins involved in the manufacture of glutathione, such as cystine/glutamate antiporter (system xc^−^) and GPX4, are recognised downstream targets of Nrf2 (Fan et al. 2017; Kerins and Ooi 2018). Of note, is that RSL-3 and erastin, which promote ferroptosis, can trigger the ferroptotic cascade through system xc^−^ and GPX4 inhibition (Dodson et al. 2019; Kong et al. 2019). In combination, the decrease in scavenging oxidation compounds, elimination of free radicals arising from the surplus iron, stimulation of the p62/Keap1/Nrf2 pathway and enhancement of nuclear Nrf2 accrual may offer possible safeguarding processes against ferroptosis following ICH (Chang et al. 2014).

A conventional form of therapy, acupuncture has been broadly acclaimed clinically for its distinctive benefits relating to the management of neurological pathologies (Schnyer et al. 2008). Scalp acupuncture (SA) has been demonstrated to be without risk and efficacious in promoting the recovery of clinical neurological impairment and limb paralysis following acute ICH (Wang et al. 2016; Song et al. 2019; Liu et al. 2014, 2019; Han et al. 2019). Li et al. (2019) evaluated the time-effect association of acupuncture on the histopathology, ultrastructure and neurological deficits associated with acute ICH in a rat model. They noted that the sooner acupuncture was initiated, the more profound the neurological recovery, particularly if commenced 3 or 9 h following ICH. Furthermore, scalp penetration acupuncture at Baihui (DU20) was shown to be a potentially efficacious treatment option for acute ICH in a meta-analysis and preclinical systematic review (Li et al. 2014).

It has been demonstrated that SA was able to enhance neurological functional recovery in a murine model of ICH (Liu et al. 2017) and that triggering of the Mincle/Syk signalling cascade could be suppressed by Baihui (DU20)-penetrating-Qubin (GB7) acupuncture, thus diminishing proinflammatory cytokine liberation (Liu et al. 2018). Apoptotic inhibition has also been recognised following acupuncture; stimulation of the Sonic hedgehog pathway has been implicated (Zhang et al. 2018a, b; Li et al. 2017a, b, c). Additionally, acupuncture may enhance nerve cell reparative abilities, an effect mediated through the inhibition of the Notch-Hes pathway, thus sustaining neural stem cell replication (Zou et al. 2015). Earlier studies have demonstrated that resistance to oxidative stress damage can be potentiated following acupuncture via Nrf2/ARE pathway stimulation (Zhao et al. 2019).

Thus, in light of previous evidence pertaining to acupuncture therapy in ICH, the aim of the current work was to evaluate the impact of SA application on ferroptosis in a murine model of ICH and to elucidate the possible pathways involved using methods including Western blot techniques, immunohistochemical staining, electron microscopy, and commercial assay kits. The hypothesis was that ferroptosis arising after ICH can be inhibited through stimulation of the p62/Keap1/Nrf2 pathway implicated in the governance of iron homeostasis and lipid peroxidation, thus mitigating the consequences of ICH-induced neurological deficits.

## Materials and Methods

### Animal Studies

All the rodents used for this study were acquired from the Laboratory Animal Centre (licence number: SYXK (Hei) 2018–015). Ethical approval for murine experimentation was obtained from the regional ethics committee at Heilongjiang University of Traditional Chinese Medicine, China (approval number: 2018–06-02–01). All animal work was performed in keeping with the eighth edition of the National Institutes of Health Guide for the Care and Use of Laboratory Animals (National Research Council of the National Academies 2011). The studies were rigorously conducted in keeping with the requisites published by the International Association for the Study of Pain (Breivik 2002). Intraperitoneal delivery of 1% pentobarbital sodium, dosed at 60 mg/kg, was used for anaesthesia. The mode of death was cervical dislocation; this ensured that the process was painless and conformed to ethical standards.

One hundred and sixty healthy male Sprague Dawley rats, weighing 280 ± 10 g and aged 8–10 weeks, were kept in living conditions characterised by a temperature of 21 ± 2 °C, relative humidity of 50 ± 5%, a 12-h diurnal cycle and unlimited availability of food and water. After 7 days of adaptive nutrition, the rodents were randomly split into five cohorts, each comprising 32 rats, i.e. control, sham, ICH model, SA therapy and the deferoxamine (DFX) (Yao et al. 2019) groups. Each cohort was subdivided into four subsets of eight rats according to the timing of sacrifice, which was performed at 6 h and 1, 3 and 7 days, respectively, following ICH induction. Bederson’s scale (Bederson et al. 1986) and haematoxylin–eosin (HE) staining were used to establish the successful outcome of the ICH model.

### ICH Model

Stereotactic delivery of autologous blood into the site of the right basal ganglia was used to generate the murine model of ICH (MacLellan et al. 2008). Following anaesthesia, as described above, the scalp was shaved. An aseptic protocol was adhered to for all surgical interventions (Liu et al. 2017). Following fixation with the stereotaxic equipment (68,001, RWD Life Science Co. Ltd., Shenzhen, Guangdong Province, China), a central incision was performed in order to reveal the bregma and coronal suture. An opening 1.0 mm in diameter was made with a dental drill at coordinates 3.5 mm and 0.2 mm lateral and posterior, respectively, to the right of the bregma (Khazipov et al. 2015). A Hamilton syringe was utilised to obtain 50 μL of autologous blood from the dorsal vein in the tail; the blood was infused at a rate of 25 μL/min and 6 mm deep into the caudate putamen. The delivery needle was extracted after a 5-min interval. Dental zinc phosphate cement was used to obturate the burr hole and the skin incision was closed with sutures. A gauze dressing was placed on the tail.

Five hours following induction of ICH, a positive model outcome was indicated by a neurological function ranking on Bederson’s scale of between 1 and 3. Only these rats were studied further. On the side opposite the cerebral lesion, elbow and wrist flexion, shoulder flexion and adduction, a diminished push-resistance to paralysis or circular motion were noted.

### Groups

The five cohorts were defined as follows, according to the intervention delivered:

(i) SA treatment group: these rats underwent SA therapy on a daily basis using a Baihui acupoint-penetrating-Qubin acupoint.
(ii) DFX cohort: 500 mg of the iron chelator DFX (Novartis Pharmaceutical Co. Ltd., Beijing, China) was dissolved in 5 mL of 0.9% saline solution and administered via intraperitoneal injection at a dose of 100 mg/kg/day. The dose was computed as described by Sun et al. (2016a, b, c).
(iii) Control group: these rats did not undergo operative intervention or receive any therapy.
(iv) Sham group: similar needle protocols were performed, but saline was administered rather than blood.
(v) ICH model cohort: these rats underwent ICH induction alone without any other interventions.

### Scalp Acupuncture Treatment

**Fig. 1:**
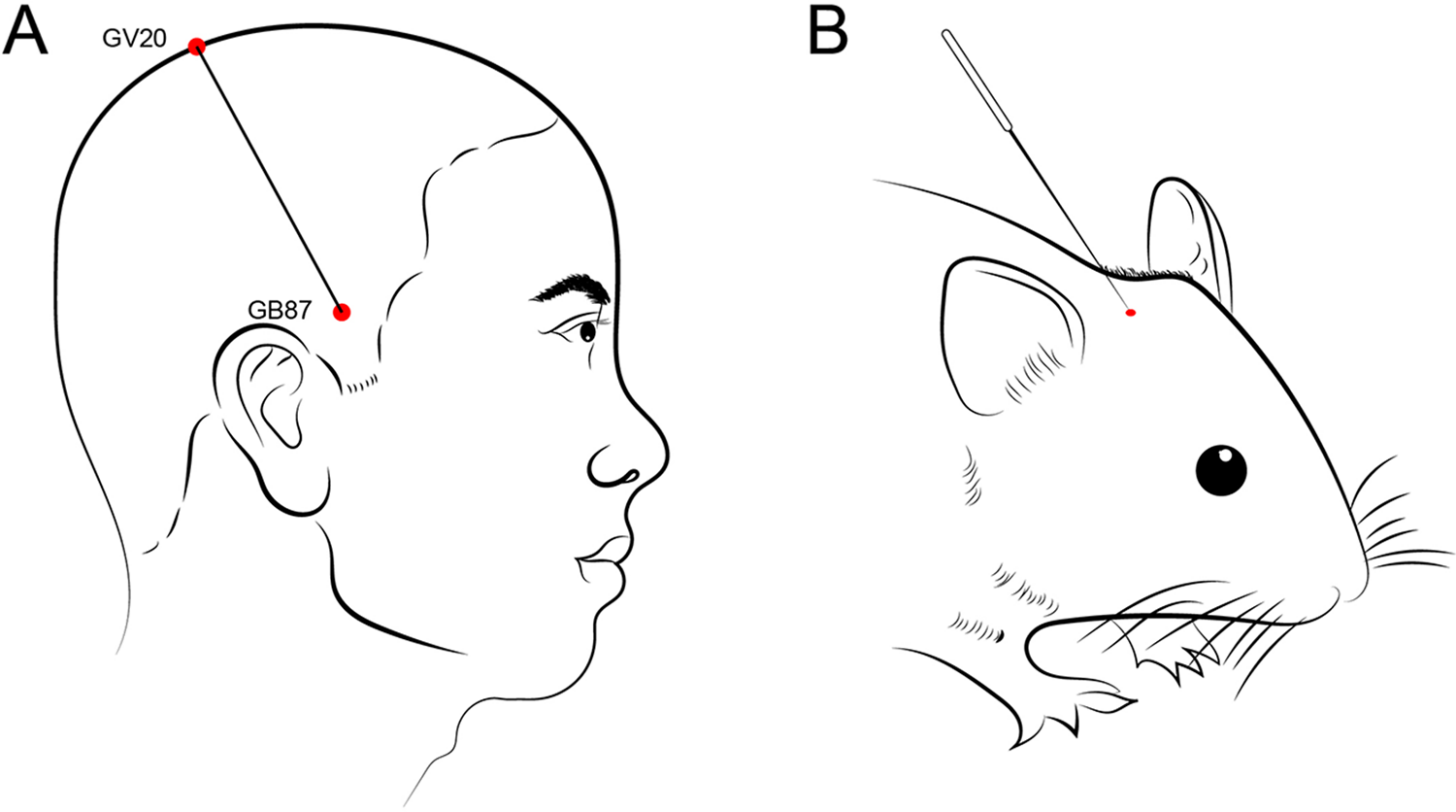
Acupuncture points in rats were determined using a transpositional method. (**A**) Location of DU20 and GB7 on humans. (**B**) Schematic diagram of DU20-GB7 on a rat. Baihui (DU20) is located at the midpoint of the line connecting the apexes of the bilateral ear on the parietal bone; Qubin (GB7) is at the posterior two thirds point of the line connecting the right orbital margin and porus acusticus externus

Rats were positioned in a rat fixator (Beijing Ji Nuotai Science and Technology Development Co. Ltd., Beijing, China). Five hours following positive identification of the generation of the model, rats in the SA cohort underwent 30 min of SA therapy with the Baihui acupoint-penetrating-Qubin acupoint on a daily basis for 7 successive days. This was performed by a qualified acupuncture practitioner and in keeping with the World Acupuncture and Moxibustion Congress transpositional method (Hua 1987; Jittiwat 2017; Yin et al. 2008) (Fig. 1).

The Baihui acupoint (DU20) was found at the point of the posterior two-thirds of the line extending from the orbital margin and its intersection with the porus acusticus externus. Sterile acupuncture needles measuring 0.30 × 25 mm (Huatuo, Suzhou Medical Supplies Factory Co. Ltd., Suzhou, China) were applied to this location, with the tip passing through the subcutaneous tissue along the vector from Baihui (DU20) to ipsilateral Qubin (GB7). Penetration was 15 mm deep and through the epicranial aponeurosis. SA was conducted at intervals of 5 min with a frequency of 180 ± 20 r/min for a total duration of 30 min. The rats from the additional experimental cohorts underwent placement on the fixator in a similar manner.

### Neurological Function Assessment

Two researchers, blinded to the study design, conducted the Ludmila Belayev test (Belayev et al. 1996; De Ryck et al. 1989) in order to score neurological impairment at 6 h and 1, 3 and 7 days, respectively, following ICH induction. Postural reflex and placement tests were assessed. The latter encompassed visual and tactile elements, both incorporating front and side, and proprioception appraisals. A scale of 0 to 2 was utilised for each; the greatest sum of deficit scores was therefore 12. The ultimate score for each test was the mean scoring designated by the two assessors. The closer the score to 12, the worse the neurological dysfunction. Further details are presented in Supplementary file 1.

### Histopathological Assay

Following conclusion of the behavioural testing, the animals were sacrificed as described above and HE staining of brain sections was performed. After perfusion with 4% paraformaldehyde, cerebral tissue samples from the peri-haemorrhagic regions were planed into 5-μm-thick sections in order to evaluate the histological features. A microscope (BX53, Olympus Corporation, Tokyo, Japan) was used at × 200 magnification for image acquisition. The findings were in keeping with the anticipated pathophysiological changes associated with cerebral haemorrhage, thus confirming that the ICH model had been effective.

### Transmission Electron Microscopy

Rodents from the sham group, together with animals at 3 and 7 days following ICH induction underwent perfusion with 2% paraformaldehyde and 2% glutaraldehyde in 0.1 M sodium cacodylate buffer, followed by post-fixing in 2% osmium tetroxide with 1.6% potassium ferrocyanide in 0.1 M sodium cacodylate (Bogacz and Krauth-Siegel 2018). Peri-haemorrhagic cerebral tissues and samples from the comparable site in the sham group were then sliced into 1 mm^3^ clumps, stained as a group with 2% uranyl acetate (UA), dehydrated in ethanol and then embedded in eponate.

Sections between 0.5 and 2 μm thick underwent HE staining; microscopy was performed to establish the alignment and precise anatomical borders of the haematoma. Subsequently, 60–90-nm-thick sections were applied to copper slot grids; 2% UA and lead citrate were utilised for staining. Micrographs were acquired using a Hitachi H-7650 transmission electron microscope (Suzhou Saiens Instrument Co., Ltd.) at the Microscopy Centre of Harbin Medical University. Ten random micrographs of the peri-haemorrhagic samples were generated for each animal from the ICH cohorts and from the equivalent site in the sham group in order to evaluate the dimensions of the mitochondria. ImageJ software (version 1.8.0, National Institutes of Health) was employed, and parameters of the entire mitochondrial area within the nerve cell body were recorded; maximal diameter was taken to reflect the longitudinal mitochondrial axis. An assessor, blinded to the intervention allocation, performed the analysis.

### Protein Extraction and Western Blot Analysis

Following deep anaesthesia and transcardial perfusion with saline, fresh cerebral samples were obtained and stored on ice, transected into coronal Sects. 4 mm thick and then maintained in cryopreservation tubes at a temperature of -80 °C prior to Western blot assays. A whole protein extraction kit (Wanleibio Biotechnology Co., Ltd., WLA019, China) and BCA protein concentration kit (Wanleibio Biotechnology Co., Ltd., WLA004, China) were employed for complete protein extraction and protein concentration assays, respectively.

Sodium dodecyl sulfate–polyacrylamide gel electrophoresis (SDS-PAGE) was utilised to segregate 30 µg protein, which was then transferred to polyvinylidene fluoride (PVDF) membranes (Millipore, Inc., IPVH00010, USA). Sixty minutes of exposure to 5% skimmed milk was used for blocking the membranes, after which they were soaked in Tris-buffered saline with 0.1% Tween-20. They were then cultured overnight at 4 °C with primary antibodies against NeuN (1:500, no. WL03099, 46 kDa, Wanleibio), Nrf2 (1:400, no. bs-1074R, 68 kDa, Bioss), FTH1 (1:1000, no. ab183781, 21 kDa, Abcam), GPX4 (1:500, no. ab125066, 22 kDa, Abcam), p62 (1:500, no. WL02385, 50 kDa, Wanleibio), Keap1 (1:500, no. WL03285, 69 kDa, Wanleibio) and β-actin (1:1000, no. WL01845, Wanleibio). The specimens were then cleansed and cultured for 45 min at 37 °C in goat-anti-rabbit IgG- horseradish peroxidase (HRP) (1:5000, no. WLA023, Wanleibio). Protein bands were uniformly encased in luminescent liquid (Wanleibio Biotechnology Co., Ltd.) and laid bare. Image scanning was conducted, and Gel-Pro-Analyzer software (version 4.0, Media Cybernetics, Inc.) was employed to assay target band optical density parameters. Normalisation of all greyscale values to those relating to β-actin was undertaken for statistical assessment.

### Immunohistochemical Staining

Once sacrificed, rats underwent decapitation, the cerebrum was extracted and tissue from peri-haemorrhagic areas was harvested from the various cohorts. Following embedding in paraffin, the specimens were sliced into 5-µm sections; xylene was utilised for dewaxing. The sections were then soaked in antigen repair solution and a low flame was applied for 10 min. After cooling to the ambient temperature, the samples underwent incubation in 3% hydrogen peroxide; goat serum was used for blocking. Threefold 5-min washing in phosphate-buffered saline was performed at each stage of the process.

The sections then underwent overnight incubation at a temperature of 4 °C with primary antibodies, i.e. rabbit anti-Nrf2 (1:200, no. bs-1074R, Bioss), rabbit anti-FTH1 (1:400, no. ab183781, Abcam) and rabbit anti-GPX4 (1:300, no. ab125066, Abcam). Incubation for 30 min at 37 °C with the equivalent secondary antibody, i.e. biotinylated goat anti-rabbit IgG (1:200, no. A0277, Beyotime) was performed. Lastly, labelling with horseradish, produced in DAB, was undertaken. The samples were soaked in distilled water and then haematoxylin counterstaining was conducted, followed by desiccation and sealing of the sections. A microscope (DP73, Olympus Corporation, Tokyo, Japan) was employed to acquire images at × 400 magnification.

### Measurement of Malondialdehyde and Iron Concentration

Malondialdehyde (MDA) (Wanleibio Biotechnology Co., Ltd., WLA048a, China) and iron (TC1015, Leagene Biotechnology Co. Ltd., China) assay kits were used in keeping with the vendor’s recommendations to determine the relative concentrations of these two factors in cerebral samples from the peri-haemorrhagic regions. MDA and iron titres were assayed at optical densities of 570 nm and 562 nm, respectively.

### Statistical Analysis

Data analysis was performed using the Statistical Package for the Social Sciences for Windows (version 26.0, IBM Corp., Armonk, NY, USA). Continuous data are given as mean ± standard deviation (mean ± SD). One-way analysis of variance (ANOVA) and Tukey’s post hoc tests were utilised to obtain *p* values for Western blotting, immunohistochemistry, and parameters of MDA and iron concentrations. Kruskal–Wallis and post hoc Dunn’s multiple comparisons tests were employed to establish *p* values for the neurological impairment scores. Histograms were generated using GraphPad Prism 8.0 software (GraphPad Software, Inc.). Statistical significance was defined as a *p* value < 0.05.

## Results

### SA Attenuates Neurobehavioural Deficits and Pathological Injury Following ICH

**Fig. 2:**
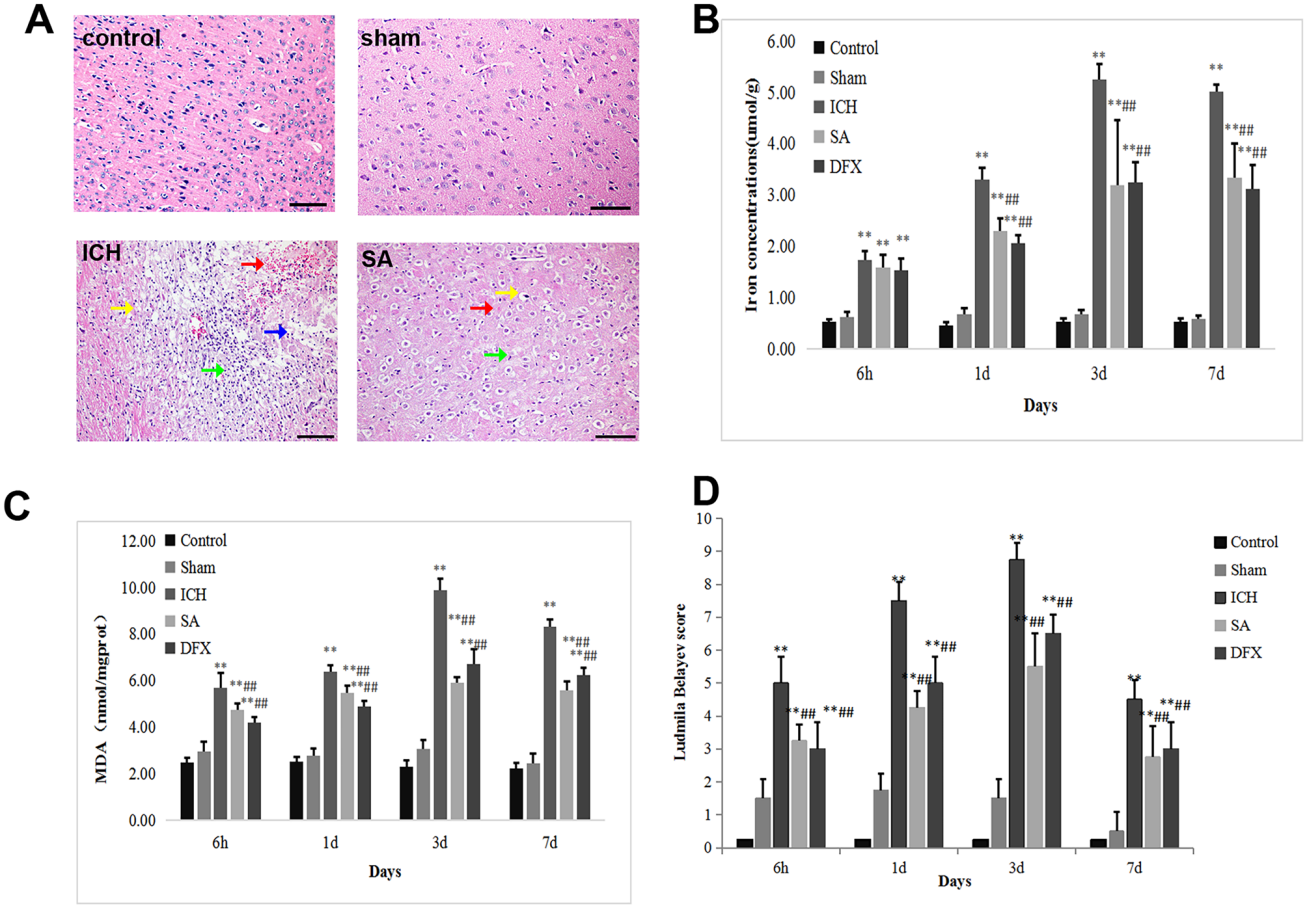
Appraisal of neurobehavioural impairment and pathological alterations after ICH. (**A**) Typical illustrations are presented in five groups from day 3 following ICH. Peri-haemorrhagic cerebral tissues of ICH rats stained with haematoxylin and eosin at × 200 magnification demonstrate blood exudation (red arrow), nuclear pyknosis and loss (blue arrow), cellular oedema and nuclear pyknosis (yellow arrow), and inflammatory cell infiltrate (green arrow). SA intervention diminished ICH-induced pathological alterations, lowering iron concentrations (**B**) and MDA (**C**), and reducing neurobehavioural deficit scores (**D**) following ICH. Data are given as mean ± SD (*n* = 8; **p < 0.05 vs. sham group; ##*p* < 0.05 vs. ICH group). Scale bar: 100 µm. ICH: intracerebral haemorrhage; SA: scalp acupuncture; DFX: deferoxamine; h: hour(s); d: day(s)

Behavioural testing was conducted at 6 h and 1, 3 and 7 days following induction of ICH in order to establish the impact of SA on neurological impairment. Similar to earlier studies (Guo et al. 2019), rats from each cohort displayed neurological deficits at 6 h after ICH; these became most dense at 3 days and were evidenced using the Ludmila Belayev scoring protocol (Fig. 2).

Histological alterations in cerebral tissue specimens from the peri-haemorrhagic areas were demonstrated with HE staining (Fig. 2A). The rats with ICH displayed a larger population of inflammatory cells and disrupted red cells. These findings were associated with tissue oedema, disorganised cellular structure, marked vacuolation of the intercellular matrix, nerve cell karyopyknosis and anachromasis, identified at day 3.

The Ludmila Belayev test scores were reduced following ICH in rats receiving DFX and SA (Fig. 2D; *p* < 0.05). The scores in the SA and DFX cohorts were equivalent (Fig. 2D). Furthermore, in these two groups, there was evident blood absorption and proportionally fewer infiltrating red cells and inflammatory cells identified. These data suggest that neurobehavioural impairment and pathological damage arising after ICH can be mitigated by SA.

### SA Treatment Attenuates Mitochondrial Morphological Damage Following ICH

**Fig. 3:**
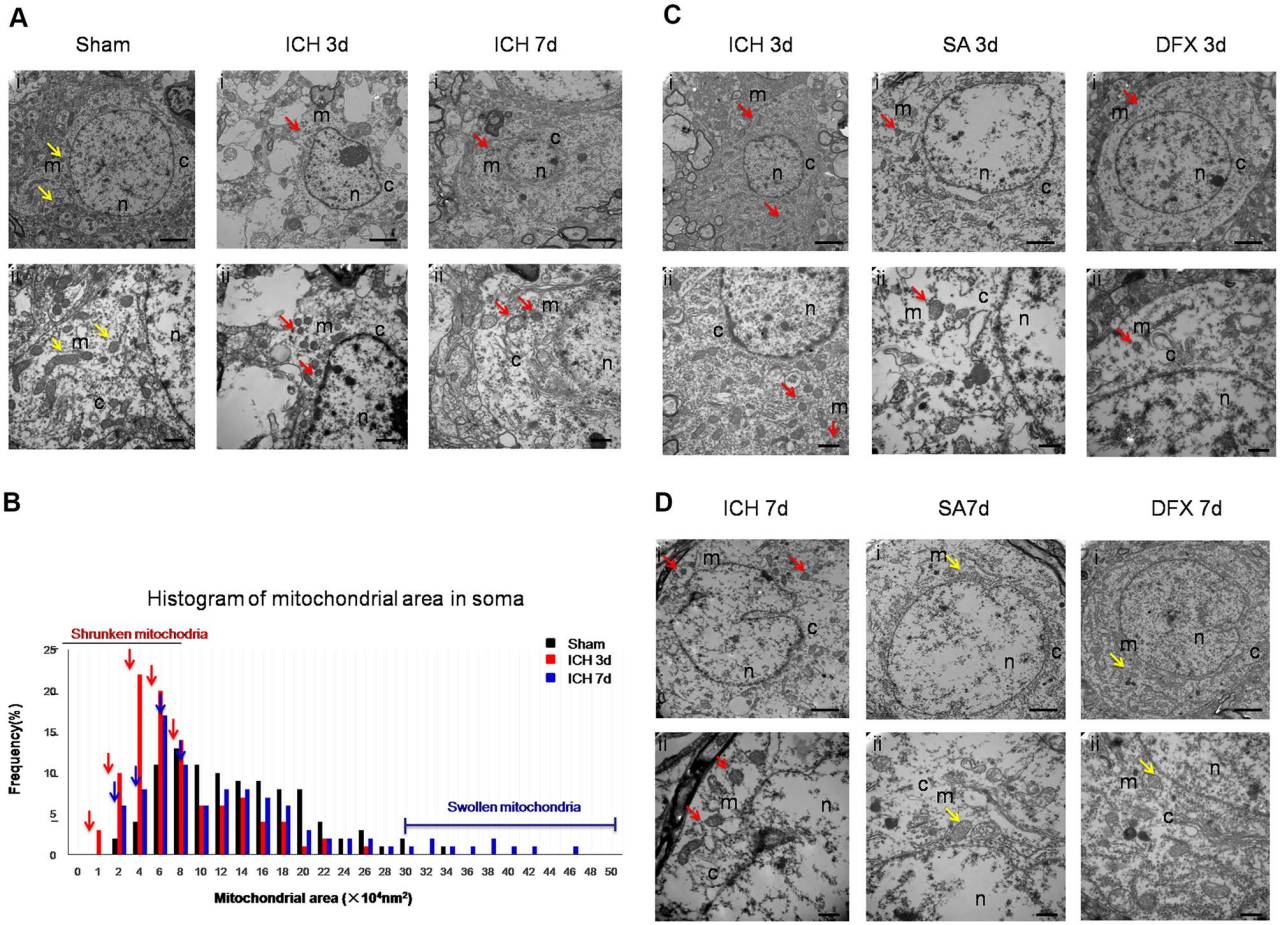
Intervention with SA mitigated ICH-induced mitochondrial structural injury. Images of nerve cell soma from haemorrhagic regions obtained from transmission electron microscopy demonstrate contracted mitochondria (red arrows) at days 3 and 7 after ICH (**A**). Ai, Ci and Di, and Aii, Cii, and Dii, were acquired at × 10,000 and × 25,000 magnification, respectively. SA diminished mitochondrial configurational abnormalities within the soma of neurons from the peri-haematoma area at day 3 (**C**) and day 7 (**D**) following ICH. Scale bars: Ai, Ci, Di, 2 µm; Aii, Cii, Dii, 500 nm. Quantification of the frequency of various mitochondrial areas in neuronal somas in the (**B**) sham group and day 3 group and day 7 group following ICH. Arrows indicate the increased frequency of shrunken mitochondria on days 3 and 7. Number of mitochondria in the soma: sham, *n* = 206; ICH day 3, *n* = 153; ICH day 7, *n* = 171. n, nucleus; c, cytoplasm; m, mitochondria; *n* = 3 animals per group

Transmission electron microscopy was used to appraise mitochondrial morphology within the nerve cell bodies in order to verify whether SA therapy diminishes ferroptosis (Li et al. 2018a, b; Lewerenz et al. 2018). Three days after ICH, the nerve cell soma demonstrated multiple shrivelled mitochondria with thickened membranes and breach of the outer membrane (Fig. 3C). There was a greater prevalence of reduced mitochondrial areas within the nerve cell bodies at day 3 and at day 7 following ICH (Fig. 3A, B). Nevertheless, when contrasted against the ICH cohort, in animals receiving SA or DFX, the nerve cell bodies exhibited dispersed contracted mitochondria with thickened and breached outer membranes at the day 3 assessment. Review at 7 days, performed to assess the full impact of SA therapy, indicated the ongoing presence of atrophic and ruptured mitochondria in the cerebral neurons from the peri-haemorrhagic regions in the rats from the ICH cohort (Fig. 3D). Interestingly, in the SA and DFX treatment groups at this juncture, the mitochondria appeared enlarged, suggesting mild nerve cell injury. These data imply that ferroptosis-associated mitochondrial structural change can be diminished by SA.

### SA Treatment Increases NeuN Expression in ICH Rats

**Fig. 4:**
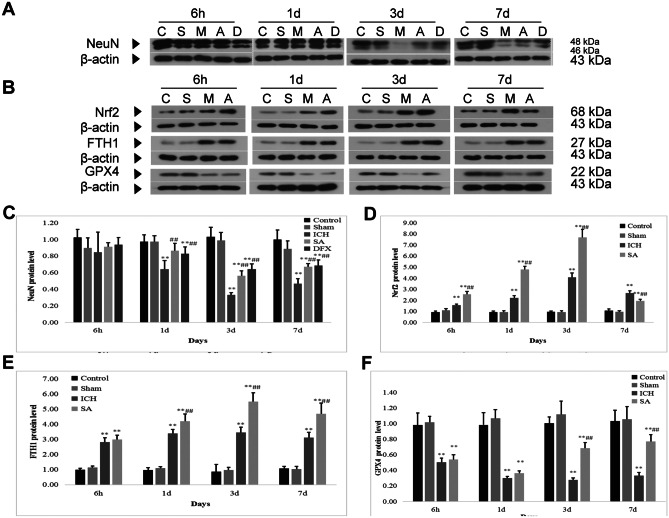
SA treatment diminished oxidative injury and reduced neuronal cell death in rats with ICH. Images illustrate level of expression and semi-quantitative data relating to NeuN (**A**, **C**), Nrf2 (**B**, **D**), FTH1 (**B**, **E**) and GPX4 (**B**, **F**) as assayed by Western blot and normalised to β-actin protein. C: control group; S: sham group; M: ICH group; A: scalp acupuncture group; D: DFX group. Data are presented as mean ± SD (*n* = 8; **p < 0.05 vs. sham group; ^##^*p* < 0.05 vs. ICH group). ICH: intracerebral haemorrhage; SA: scalp acupuncture; DFX: deferoxamine; Nrf2: nuclear factor erythroid 2-related factor 2; GPX4: glutathione peroxidase 4; FTH1: ferritin heavy chain 1; h: hour(s); d: day(s)

NeuN expression was established using Western blot assay to affirm that nerve cells were susceptible to damage following ICH (Fig. 4A). In contrast to specimens obtained from the sham cohort, marked neuronal demise was seen at 1, 3 and 7 days, respectively, after ICH. However, both DFX and SA interventions appeared to salvage nerve cells in these contexts. NeuN expression was notably greater in the SA and DFX therapy cohorts (Fig. 4C; *p* < 0.05), but comparable between these two intervention groups (Fig. 4C).

### SA Treatment Alleviates ICH-Induced MDA Accumulation by Increasing GPX4 Expression

Figure 2C illustrates the absorbance data and demonstrates that peri-haematomal areas within the ICH rats contained higher titres of MDA in contrast to the sham cohort (*p* < 0.05). Intervention with either SA or DFX diminished the MDA concentration in peri-haemorrhagic tissues compared with the ICH cohort, but these two treatment groups exhibited comparable concentrations (Fig. 2C). These results suggest that SA mitigates the accrual of MDA in rats with induced ICH.

**Fig. 5:**
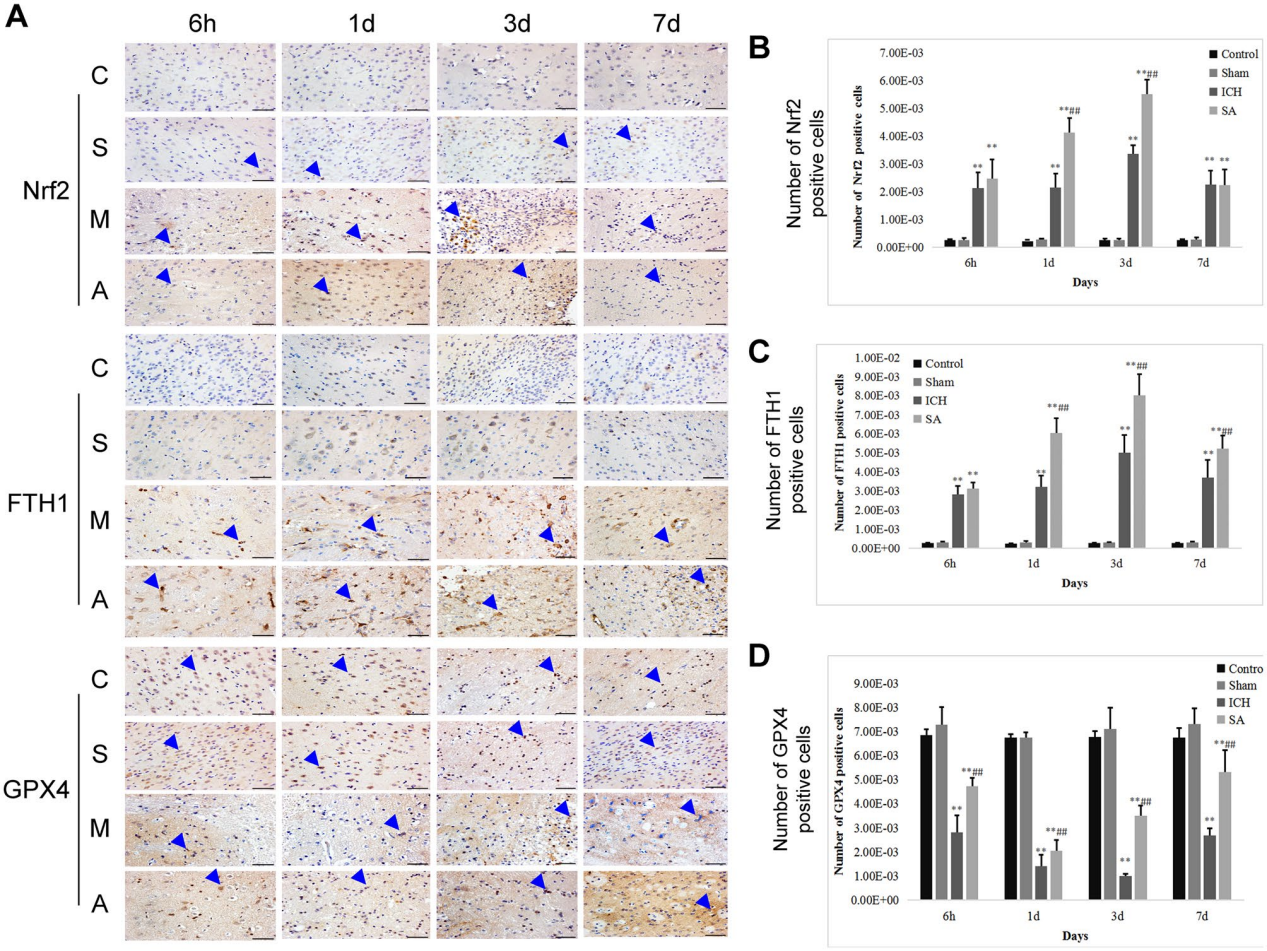
SA therapy enhanced nuclear Nrf2 accretion and attenuated oxidative injury after ICH. The frequency of cells, counted at × 200 magnification, positive (arrows) for Nrf2, GPX4 and FTH1 in the peri-haematomal region from the basal ganglia as characterised by immunohistochemistry (**A**). Scale bars: 100 µm. C: control group; S: sham group; M: ICH group; A: scalp acupuncture group. Semi-quantitative analysis is shown for cells positive for Nrf2 (**B**), FTH1 (**C**) and GPX4 (**D**). Data are presented as mean ± SD (*n* = 8; **p < 0.05 vs. sham group; ^##^*p* < 0.05 vs. ICH group). ICH: intracerebral haemorrhage; SA: scalp acupuncture; Nrf2: nuclear factor erythroid 2-related factor 2; GPX4: glutathione peroxidase 4; FTH1: ferritin heavy chain 1; h: hour(s); d: day(s)

Oxidative stress is a major component of secondary damage following ICH (Zhang et al. 2018a, b). GPX4 is a key participant in oxidative stress equilibrium (Li et al. 2018a, b); following ICH, GPX4 titres are diminished, thus aggravating the insult caused by ferroptosis lipid peroxidation (Wu et al. 2013; Wenzel et al. 2017). In order to establish the association between the accretion of GPX4 and MDA in the ICH model, GPX4-positive cells were identified. Immunopositivity for GPX4 implied that GPX4 levels diminished 6 h and 3 days following ICH but rose following therapy with SA (Fig. 5A, D; *p* < 0.05). According to the Western blot results, upregulation of GPX4 expression was present following SA when judged against the ICH cohort for the various time junctures (Figs. 4B and 5F; *p* < 0.05). In combination, these data infer that SA may diminish the oxidative stress damage precipitated by ICH by amplifying GPX4 expression.

### SA Treatment Alleviates ICH-Induced Iron Accumulation by Increasing FTH1 Expression

Figure 2B depicts the ongoing increase in total iron concentration in peri-haemorrhagic areas following ICH, with a zenith at day 3 and continuing elevation at day 7 when judged against the sham cohort (*p* < 0.05). In contrast to the ICH model animals, therapy with SA and DFX diminished iron concentrations (Fig. 2B; *p* < 0.05), but the iron titres within samples from the latter two intervention groups were equivalent (Fig. 2B). These data imply that iron accrual within rats with induced ICH is mitigated by SA.

Several researchers have demonstrated that the iron-sequestering protein, FTH1, is involved in the governance of iron homeostasis following ICH (Yang et al. 2016; Bogdan et al. 2016). FTH1 expression was therefore investigated in order to elucidate the processes that contribute to the attenuation of surplus iron accumulation. In contrast to the sham cohort, elevated iron titres were present at all the studied time points following ICH (Fig. 2B; *p* < 0.05). There was an equivalent rise in FTH1-positive cells at these junctures in rats with ICH. In contrast to the ICH cohort, in the SA therapy rats, the prevalence of FTH1-positive cells was raised (Fig. 5A, C; *p* < 0.05), and iron titres were diminished. Western blot was used to affirm the association between FTH1 and iron sequestration. Cerebral FTH1 protein levels were greater at the time intervals evaluated after SA therapy than in the ICH rats (Fig. 4B, E; *p* < 0.05). The data, therefore, indicate that intervention with SA may augment FTH1 expression which, in turn, diminishes iron accretion caused by ICH.

In order to determine whether the enhanced degree of FTH1 was involved in mitigating nerve cell lipid peroxidation damage induced by surplus iron, MDA was assayed at day 3 following ICH, i.e. the time of greatest oxidative insult. Iron accrual was at its height at this time point. In combination, these data infer that SA treatment is efficacious in reducing nerve cell lipid peroxidation damage as a result of excess iron through the amplification of FTH1 expression.

### SA Treatment may Increase FTH1 and GPX4 Levels by Enhancing Nuclear Accumulation of Nrf2

Within the context of oxidative stress, Nrf2 is an essential moderator (Kang and Tang 2017); its vital downstream proteins include GPX4 and FTH1. In order to determine whether SA therapy elevates FTH1 and GPX4 concentrations via nuclear Nrf2 accretion, immunohistochemical techniques were utilised to assay cerebral tissue Nrf2 immunopositivity. In contrast to the ICH cohort, the rats receiving SA demonstrated a greater number of Nrf2-positive cells; nuclear accrual of Nrf2 was higher on days 1 and 3 following ICH (Fig. 5A, B; *p* < 0.05). Concomitantly, the immunopositivity data with respect to GPX4 and FTH1 were in keeping with the changes in the Nrf2 titres (Fig. 5A, C, D). Western blotting was used to assay the degree of protein expression of both Nrf2 and the downstream transcripts, GPX4 and FTH1; the results supported the immunopositivity data (Fig. 4B, D-F). These findings indicate that FTH1 and GPX4 levels are elevated by SA via the promotion of nuclear Nrf2 accretion.

### SA Treatment Promotes Nrf2 Transcription by Enhancing the Interaction Between p62 and Keap1

**Fig. 6:**
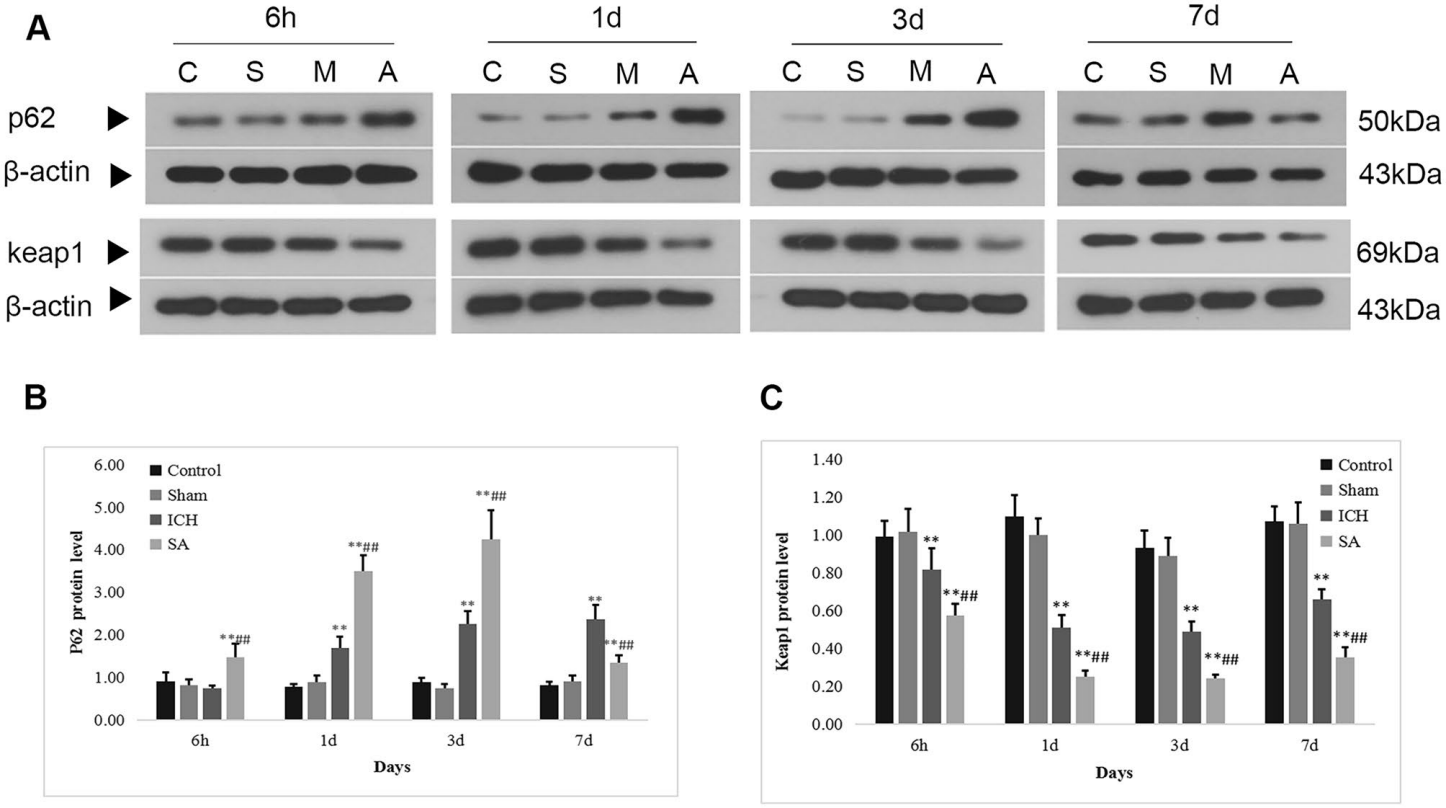
SA therapy enhanced the p62/Keap1/Nrf2 pathway in rats with induced ICH. Western blotting analysis of p62 and Keap1 expression, normalised to β-actin protein (**A**). C: control group; S: sham group; M: ICH group; A: scalp acupuncture group. Quantitative analysis of p62 protein expression (**B**) and Keap1 protein expression (**C**). Data are presented as mean ± SD (*n* = 8; **p < 0.05 vs. sham group; ^##^*p* < 0.05 vs. ICH group). ICH: intracerebral haemorrhage; SA: scalp acupuncture; p62: sequestosome 1 (p62/SQSTM1); Keap1: Kelch-like ECH-associated protein-1; h: hour(s); d: day(s)

Contemporary work has shown that in the context of stress, such as hepatic injury (Shen et al. 2018) or in head and neck malignancy (Roh et al. 2017), the substrate adaptor p62 protein, also referred to as sequestosome 1, has an immediate impact on Nrf2 expression by attaching to Keap1. In order to further appreciate the pathways involved in the upregulation of Nrf2 following ICH, Western blot was utilised to assay the degree of expression of p62 and Keap1 proteins (Fig. 6A–C). In cerebral samples from the rats with ICH, p62 expression was elevated and Keap1 protein expression was diminished. Furthermore, in contrast to the ICH cohort, rats receiving SA therapy demonstrated heightened expression of p62 protein and reduced Keap1 protein expression on days 1 and 3, respectively, following ICH (Fig. 6B, C; *p* < 0.05). The possibility of p62 attaching to Keap1 so as dislodge Nrf2, to suppress Nrf2 breakdown and to promote nuclear accretion, was then explored. Western blot data revealed a marked rise in Nrf2 titres in the immediate phase concomitant with the rise and fall in p62 and Keap1 concentrations, respectively (Fig. 4B, D; *p* < 0.05). All these findings imply that the engagement of p62 and Keap1 underlies the nuclear amassment of Nrf2 in ferroptosis, and that therapy with SA may augment Nrf2 transcription by promoting the association between p62 and Keap1.

## Discussion

ICH is a grave and catastrophic pathology. Despite the fact that current treatment approaches which target the presently established pathways relating to cell necrosis have attained positive clinical endpoints, a large number of patients experience residual neurological deficits following the cerebrovascular event (Rocha et al. 2020).

Lately, ferroptosis has been the subject of attention as it is a type of programmed cell demise that is implicated in malignancy, ICH, reperfusion damage following ischaemia and renal dysfunction, amongst additional pathologies (Stockwell et al. 2017; Sarhan et al. 2018). Both iron and lipid pathways are tightly linked to the disease processes underlying ICH. Earlier work has demonstrated that iron, a major component of haemoglobin, which is extremely toxic to nerve cells, is liberated during haematoma formation following ICH and can initiate a Fenton reaction. The latter leads to the generation of multiple hydroxyl radicals, which stimulate and exacerbate tissue oxidation which harms cellular membranes, DNA and proteins and gives rise to nerve cell ferroptosis (Li et al. 2017a, b, c; He et al. 2020; Magtanong and Dixon 2018; Zhang et al. 2018a, b; Djulbegovic and Uversky 2019).

In the current research, injury from lipid peroxidation was recognised via the iron and MDA titres, which implied that oxidative stress products progressively increased and attained a zenith at day 3 after ICH. Proof of ferroptosis was identified by demonstrating the mitochondrial morphological characteristics particular to ferroptosis in the nerve cell bodies of cerebral tissue collected from the peri-haemorrhagic regions. Such traits included ridge attenuation, breach of the outer membrane and diminished dimensions. These findings support the presence of active ferroptosis in haemorrhagic cerebral tissue (Dixon et al. 2012).

It was also determined that therapy with SA attenuated the neurological deficits which arise after ICH by suppressing ferroptosis in a manner akin to the prophylactic activity of the iron chelator, DFX. This acts through the reduction of surplus iron thus protecting against injury via lipid peroxidation (Kontoghiorghe and Kontoghiorghes 2016; Hu et al. 2019). In particular, evidence has been presented that following ICH, intervention with SA safeguards the cerebral tissue through promotion of the p62/Keap1/Nrf2 antioxidant signalling pathway, which is a major governor of ferroptosis via transcriptional stimulation of Nrf2, which is implicated in reactive oxygen species (ROS) and iron homeostasis (Tan et al. 2020).

Nrf2 is a key moderator within the context of oxidative stress and acts as an antioxidant in safeguarding cells against ferroptosis (Abdalkader et al. 2018). Earlier research has demonstrated that activation of Nrf2 and the Nrf2-governed antioxidant pathways can moderate ROS and NF-κB, triggering the NLRP3 inflammasome pathway and thus diminishing the acute cerebral insult in male Sprague–Dawley murine models of ICH (Zhao et al. 2007; Zeng et al. 2017). This is also supported by the findings that Nrf2 knockout mice sustain a larger sized injury at 3 days following induced ICH and exhibit a higher prevalence of iron-rich cells compared to the wild type equivalents (Chang et al. 2014).

Normally, Nrf2 exists in an inactivated form via Keap1-moderated ubiquitination and breakdown within the proteasome (Fan et al. 2017; Toyokuni 2014). The current work showed that normal cerebral tissue exhibits a low degree of Nrf2 expression. Nrf2 titres progressively rose, attaining their maximum value when iron excess and oxidative injury were at their worst on day 3 after ICH. The heightened nuclear accrual of Nrf2 at the same time point was demonstrated using immunohistochemical techniques. A range of proteins responsible for detoxification and antioxidant effects are encoded for by Nrf2, such as GPX4 (Fischer et al. 2019).

Yang et al. (2014) have noted that GPX4 is a mammalian selenoprotein glutathione peroxidase which acts as an essential participant in lipid reparation following oxidative injury. Additional researchers have demonstrated that GPX4 deficiency stimulated spinal cord degeneration of motor neurons, a process typified by ferroptosis, thus illustrating that GPX4 is a prerequisite for motor neuron longevity (Chen et al. 2015). The present work reported that with the worsening of oxidative stress damage, GPX4 levels progressively declined (Zhang et al. 2018a, b). At its nadir, on day 3 following ICH, notable alterations in the mitochondria were evident, suggestive of ferroptosis. Additional research has offered data to indicate that genetic overexpression of GPX4 leads to a rise in GPX4 levels, attenuated nerve cell impairment and oxidative stress after a haemorrhagic cerebral event. On the contrary, using specific pharmaceutical inhibitory agents to block GPX4 or in GPX4 genetic knockout models, severe brain injury (SBI) was aggravated following ICH (Zhang et al. 2018a, b).

Nrf2 also governs FTH1, which is another crucial gene. Ferritin is an essential cerebral binding protein; it comprises two subunits, FTH1 and FTL. FTH1 contains ferrous oxidase activity and principally contributes to the swift absorption and re-use of iron, whereas FTL essentially assists in sustaining the integrity of ferritin, which is associated with the chronic ability of cells to harbour iron (Liu et al. 2013).

Murine studies have demonstrated that in ICH models as opposed to sham cohorts, FTH1 levels were notably elevated following ICH (Yang et al. 2016). The observations from the present study are in agreement; FTH1 expression in normal cerebral tissues was low, but progressively rose together with enhanced iron sequestration following ICH. Additionally, it was reported that SA therapy may amplify FTH1 expression, thus augmenting iron absorption and re-use as indicated by diminished iron titres and elevated FTH1 levels in the SA cohort on day 3 after ICH. Of note is that the patterns of FTH1 and GPX4 level changes corresponded to the fluctuation of Nrf2 concentrations. Nrf2 stimulation may be essential to offset the impact of diminished GPX4 activity (Kerins et al. 2018), which is equivalent to Nrf2 knockout. Sensitivity to ferroptosis is heightened by FTH1 knockout (Sun et al. 2016a, b, c; Fan et al. 2017). The previously discussed studies reinforce the concept that Nrf2 might be a dependent transcription factor which moderates GPX4 and FTH1 expression. In the current study, SA appears to enhance TFH1 and GPX4 expression via a Nrf2-dependent pathway, thus also augmenting the absorption and use of surplus iron, diminishing iron excess and mitigating ongoing lipid peroxidation damage.

The mechanisms underlying nuclear Nrf2 accretion were also investigated. Earlier work has demonstrated that p62 acts as a multi-purpose scaffold protein that participates in a range of signal transduction pathways (Sánchez-Martín and Komatsu 2018). Keap1-dependent degradative ubiquitination is a process that controls Nrf2 (Hassannia et al. 2018). However, during oxidative stress, p62 engages with Keap1’s binding site for Nrf2, thus competitively preventing the association between Keap1 and Nrf2 and generating nuclear Nrf2 accrual (Xie et al. 2016).

It has also been demonstrated that the p62/Keap1/Nrf2 antioxidant signalling pathway safeguards cells with respect to ferroptosis (Sun et al. 2016a, b, c; Fischer et al. 2019). It was, therefore, postulated that Nrf2 detaches from its binding site on Keap1 and migrates to the nucleus, thus associating with factors pertaining to the antioxidant response of target genes in relation to several forms of damage. Consistent with this theory, Western blot data (Figs. 4 and 6) demonstrated that Nrf2’s antioxidant influence coexisted with a rise and fall in p62 and Keap1 expression, respectively. Furthermore, the engagement of p62 and Keap1 can be promoted by SA therapy, thus augmenting nuclear Nrf2 accretion and heightening its antioxidant activity.

The control of iron metabolism and stimulation of antioxidant pathways in order to suppress ferroptosis is an encouraging therapeutic goal for future ICH therapy. However, additional research is needed to elucidate the functional alterations and mechanisms underlying ferroptosis at a molecular level. Evidence has been presented with respect to the way in which SA therapy safeguards against nerve cell ferroptosis lipid peroxidation in a murine model of ICH. Nevertheless, additional work utilising specific gene knockout murine models, such as Nrf2 knockout, or pertinent inhibitors may add weight to these findings, thus offering unequivocal proof that SA can give rise to Nrf2-dependent FTH1 and GPX4 expression in cerebral tissues following ICH. In addition, in vitro experiments in models of ferroptosis to investigate the impact of Nrf2 stimulation could further elucidate the process. In answering this question, the current data will offer an essential basis for future ICH therapy focusing on the processes relating to cellular demise.

In summary, the current research recognised the part played by ferroptosis in a murine model of ICH and offered evidence to suggest that therapy with SA safeguards cerebral tissue against immediate ferroptosis lipid peroxidation damage via its action on the p62/Keap1/Nrf2 pathway antioxidant system, thus resulting in enhanced nuclear accrual of Nrf2. The prophylactic influence of SA may arise through a moderating effect on the engagement of the p62 and Keap1 proteins. This process may amplify FTH1 expression stimulating the use of surplus iron and triggering activity of the antioxidant enzyme, GPX4 which, in turn, augments oxidation product eradication. Moreover, SA therapy, in keeping with DFX, can lead to the removal of surplus iron, diminish the injury arising from lipid peroxidation and encourage the functional recovery of neurological impairment. These results add to the current literature by proposing that the influence of SA on iron metabolism and lipid peroxidation may be one pathway through which SA promotes positive clinical endpoints after ICH.

## Supplementary Information

Below is the link to the electronic supplementary material.

Supplementary file1 (DOCX 14 KB)

Supplementary file2 (DOCX 14 KB)

Supplementary file3 (DOC 18 KB)

## Data Availability

The data sets used and/or analysed during the current study are available from the corresponding author on reasonable request.
