# Supplementary material for: Scalp Acupuncture Protects Against Neuronal Ferroptosis by Activating The p62-Keap1-Nrf2 Pathway in Rat Models of Intracranial Haemorrhage
Source: J Mol Neurosci. 2021 Aug 17;72(1):82–96. doi: 10.1007/s12031-021-01890-y (PMC8755669; doi:10.1007/s12031-021-01890-y)
Supplement: Supplementary file 1 — Supplementary file1 (DOCX 14 KB) [file 12031_2021_1890_MOESM1_ESM.docx]

Ludmila Belayev Scoring Method

| Item | Score |
| --- | --- |
| 1. Postural reflex: Lift rat tail by hand and suspend rat at a height of 10 cm from the desktop.The normal reaction was bilateral anterior claw opening. |  |
| 1.1No neurological deficit | 0 points |
| 1.2Contralateral anterior limb flexion of cerebral hemorrhage focus | 1 points |
| 1.3Besides1.2, the resistance decreases of the side push table on the flexion side of the forelimb . | 2 points |
| 2. Placement test |  |
| 2.1Visual test |  |
| Front of the body: Grasp the rat with hand, expose its forelimbs, and the front side of the rat body face the desktop directly. Keep the rat at a position of 10 cm away from the edge of the table, and move the rat slowly to the desktop. |  |
| 2.1.1Rat's forepaw grasps the table quickly | 0 points |
| 2.1.2The reaction was delayed, but the time was less than 2 seconds. | 1 points |
| 2.1.3The reaction was delayed, but the time was longer than 2 seconds; or there was no response. | 2 points |
| Side of the body: Grasp the rat with hand, expose its forelimb,the left side of the rat body face the table and keep the rat at a distance of 10 cm from the edge of the table. Slowly move the rat to the edge of the table |  |
| 2.1.1The left forepaw of the rat grabbed the table quickly | 0 points |
| 2.1.2The reaction was delayed, but the time was less than 2 seconds | 1 points |
| 2.1.3The reaction was delayed, but the time was longer than 2 seconds; or there was no response. | 2 points |
| 2.2Tactile test |  |
| Front of the body：The rat's eyes are completely covered, and the rat's forelimbs are exposed. The front side of the rat body face the desktop .The forepaw touches the edge of the table gently. The stimulation depth reaches only the skin and hair, and the whisker can't touch the desktop. |  |
| 2.2.1Rat's forepaw grasps the table quickly | 0 points |
| 2.2.2The reaction was delayed, but the time was less than 2 seconds. | 1 points |
| 2.2.3The reaction was delayed, but the time was longer than 2 seconds; or there was no response. | 2 points |
| Side of the body：The rat's eyes are completely covered, and the rat's forelimbs are exposed. The left side of the rat body face the table. The left forepaw touches the edge of the table gently. The stimulation depth reaches only the skin and hair, and the whisker can't touch the desktop. |  |
| 2.2.1Rat's forepaw grasps the table quickly | 0 points |
| 2.2.2The reaction was delayed, but the time was less than 2 seconds. | 1 points |
| 2.2.3The reaction was delayed, but the time was longer than 2 seconds; or there was no response. | 2 points |
| 2.3Proprioceptive test：The rat's eyes are completely covered, and the rat's forelimbs are exposed. The front side of the rat body face the desktop. The forepaw touches the edge of the table gently. The stimulation depth reaches muscles and joints |  |
| 2.3.1Rat's forepaw quickly developed strong resistance to the desktop | 0 points |
| 2.3.2The resistance of rat forepaw to desktop was weakened and the response was delayed, but the time was less than 2 seconds. | 1 points |
| 2.3.3Rats'forepaws showed little or no resistance to the desktop and delayed response for more than 2 seconds; or no response. | 2 points |
