# Supplementary material for: Scalp Acupuncture Protects Against Neuronal Ferroptosis by Activating The p62-Keap1-Nrf2 Pathway in Rat Models of Intracranial Haemorrhage
Source: J Mol Neurosci. 2021 Aug 17;72(1):82–96. doi: 10.1007/s12031-021-01890-y (PMC8755669; doi:10.1007/s12031-021-01890-y)
Supplement: Supplementary file 2 — Supplementary file2 (DOCX 14 KB) [file 12031_2021_1890_MOESM2_ESM.docx]

**Scalp Acupuncture Protects against Neuronal Ferroptosis by Activating the p62-Keap1-Nrf2 Pathway in ICH Rat models**

**Ming-Yue Li^1^, Xiao-Hong Dai^1^, Xue-Ping Yu^1^, Wei Zou^1, 2,*^, Wei Teng^1^, Peng Liu^1^, Xin-yang Yu^2^, Qi An^3^, Xin Wen^3^**

^1^Department of Neurology, First Affiliated Hospital, Heilongjiang University of Chinese Medicine, Harbin, Heilongjiang Province, China

^2^Clinical Key Laboratory of Integrated Traditional Chinese and Western Medicine, Heilongjiang University of Chinese Medicine, Harbin, Heilongjiang Province, China

^3^Heilongjiang University of Chinese Medicine, Harbin, Heilongjiang Province, China

*** Correspondence:**

Wei Zou

[kuangzou1965@163.com](mailto:kuangzou1965@163.com)

**Author Contributions**

MYL and WZ designed the experiments. MYL, QA, and XW carried out the experiments. XHD and XPY collected the data. WT, PL, and XYY analyzed the data. MYL, XHD, and PL wrote the manuscript. XYY, XPY, and WZ revised the manuscript. All authors approved the final version of the paper.

**Conflicts of Interest**

None declared.

**Financial support**

This study was supported by the National Natural Science Foundation of China (Grant No. 81473764,81273824). The funding body played no role in the study design; collection, analysis, and interpretation of data; writing of the paper; and decision to submit the paper for publication.

**Institutional review board statement:**

The study was approved by the Animal Ethics Committee of Heilongjiang University of Chinese Medicine of China (approval No. 2018-06-02-01).
