# Supplementary material for: Scalp Acupuncture Protects Against Neuronal Ferroptosis by Activating The p62-Keap1-Nrf2 Pathway in Rat Models of Intracranial Haemorrhage
Source: J Mol Neurosci. 2021 Aug 17;72(1):82–96. doi: 10.1007/s12031-021-01890-y (PMC8755669; doi:10.1007/s12031-021-01890-y)
Supplement: Supplementary file 3 — Supplementary file3 (DOC 18 KB) [file 12031_2021_1890_MOESM3_ESM.doc]

*Journal of Molecular Neuroscience*

Dear Editor:

I wish to submit an Original Article for publication in *Journal of Molecular Neuroscience*

titled “Scalp Acupuncture Protects against Neuronal Ferroptosis by Activating the p62-Keap1-Nrf2 Pathway in ICH Rat models”. The paper was coauthored by Ming-Yue Li, Xiao-Hong Dai, Xue-Ping Yu, Wei Teng, Peng Liu, Xin-yang Yu, Qi An, and Xin Wen.

This study sought to investigate the effects of scalp acupuncture (SA) treatment on neurological function and elucidate whether SA treatment could alleviate secondary injury after intracerebral hemorrhage (ICH) by regulating ferroptosis, alongside analyzing its underlying mechanisms. Results demonstrated that SA treatment could attenuate neurological functional impairment of ICH rats, according to the Ludmila Belayev test, and improve pathological injury according to hematoxylin-eosin staining. Further, results also revealed that SA treatment could attenuate neuronal ferroptosis in ICH rats. We believe that our study makes a significant contribution to the literature because, taken together, results overall suggested that the p62/Keap1/Nrf2 signaling pathway is responsible for alleviating ferroptosis lipid peroxidation injury after ICH, thereby providing new clues with regards to SA treatment of ICH.

Further, we believe that this paper will be of interest to the readership of your journal because of its clinical implications.

This manuscript has not been published or presented elsewhere in part or in entirety and is not under consideration by another journal. All experimental procedures were approved by the ethics committee of Heilongjiang University of Traditional Chinese Medicine of China (approval No. 2018-06-02-01). We have read and understood your journal’s policies, and we believe that neither the manuscript nor the study violates any of these. There are no conflicts of interest to declare.

Thank you for your consideration. I look forward to hearing from you.

Sincerely,

Wei Zou

[Department of Neurology, First Affiliated Hospital, Heilongjiang University of Chinese Medicine, Harbin, Heilongjiang Province, China]

Email: [kuangzou1965@163.com](mailto:kuangzou1965@163.com)
